# Use of Transposon Directed Insertion-Site Sequencing to Probe the Antibacterial Mechanism of a Model Honey on *E. coli* K-12

**DOI:** 10.3389/fmicb.2021.803307

**Published:** 2022-01-17

**Authors:** Maria Masoura, Mathew T. Milner, Tim W. Overton, Konstantinos Gkatzionis, Peter A. Lund

**Affiliations:** ^1^School of Chemical Engineering, University of Birmingham, Birmingham, United Kingdom; ^2^Institute of Microbiology and Infection (IMI), University of Birmingham, Birmingham, United Kingdom; ^3^Department of Food Science and Nutrition, School of the Environment, University of the Aegean, Lemnos, Greece

**Keywords:** TraDIS, genome wide mutagenesis, flow cytometry, antimicrobial, mechanism, model, honey

## Abstract

Antimicrobial resistance is an ever-growing health concern worldwide that has created renewed interest in the use of traditional anti-microbial treatments, including honey. However, understanding the underlying mechanism of the anti-microbial action of honey has been hampered due to the complexity of its composition. High throughput genetic tools could assist in understanding this mechanism. In this study, the anti-bacterial mechanism of a model honey, made of sugars, hydrogen peroxide, and gluconic acid, was investigated using genome-wide transposon mutagenesis combined with high-throughput sequencing (TraDIS), with the strain *Escherichia coli* K-12 MG1655 as the target organism. We identified a number of genes which when mutated caused a severe loss of fitness when cells were exposed to the model honey. These genes encode membrane proteins including those involved in uptake of essential molecules, and components of the electron transport chain. They are enriched for pathways involved in intracellular homeostasis and redox activity. Genes involved in assembly and activity of formate dehydrogenase O (FDH-O) were of particular note. The phenotypes of mutants in a subset of the genes identified were confirmed by phenotypic screening of deletion strains. We also found some genes which when mutated led to enhanced resistance to treatment with the model honey. This study identifies potential synergies between the main honey stressors and provides insights into the global antibacterial mechanism of this natural product.

## Introduction

The use of honey to treat infections dates back several millennia, and with the growing global threat of AMR, natural products such as honey are being explored as alternatives to antibiotics. Although the antimicrobial activity of various honeys has been extensively studied, to date the mechanisms by which honey acts are still not fully understood (Albaridi, 2019). The high variability of honey composition makes it difficult to draw general conclusions about mechanisms, and it is likely that the effects of honey arise from synergistic interactions between several different components (Brudzynski and Sjaarda, 2021). A fuller understanding of the antibacterial mechanism of honey would be helpful in promoting the acceptance of this product as an alternative form of antimicrobial treatment and could also enable the development of standardized model honeys and medicinal honey formulations.

Regardless of the physicochemical variability between different honeys, enzymatic conversion of glucose to gluconate and hydrogen peroxide (H_2_O_2_) is believed to have a major role in the antimicrobial activity of honey (Brudzynski et al., 2017; Brudzynski, 2020; Masoura et al., 2020). This is mediated upon honey dilution by the bee-derived enzyme glucose oxidase (GOx). Both gluconic acid and H_2_O_2_ act as antibacterial/antifungal agents (Bucekova et al., 2014). Other phytochemical components (e.g., antimicrobial peptides, polyphenols, transition metals etc.), and acidity, all of which vary depending on the floral source of the nectar, can enhance the antimicrobial strength of different honeys (Sindi et al., 2019). The autooxidation of polyphenols in the presence of transition metals such as Fe (II) and Cu (I) can further enhance the production of H_2_O_2_. Also, a high correlation was found between honey color, polyphenols and/or total flavonoids, minerals and the acidity of honey. The binding of metal and protein to polyphenols increases in acidic conditions. Polyphenols can be involved in binding of proteins, carbohydrates, and lipids of bacterial cell membranes, which then exerts antibiofilm forming activity and inhibition of bacterial growth (Sindi et al., 2019). Thus, darker honeys (e.g., from buckwheat pollen), which are usually more abundant in polyphenol content, demonstrate higher antimicrobial activity. These honeys also usually result in a higher concentration of H_2_O_2_ upon dilution, which increases the generation of hydroxyl radicals (Bucekova et al., 2018; Brudzynski, 2020).

The damage caused by different kinds of model honey including model honeys has been further studied using a range of methods including microscopy (TEM, SEM), flow cytometry (FC), and screening of single gene knockouts. TEM micrographs showed enlargement of both Gram-negative and Gram-positive bacteria which might be due to partial disruption or degradation of the cell wall, which in turn could lead to the formation of spheroplasts, cell lysis and cytoplasmic leakage (Nishio et al., 2016). Two types of H_2_O_2_-producing acacia honeys were reported to significantly reduce the viability of *H. influenzae* (Newby et al., 2018) and enterohemorrhagic *Escherichia coli* O157:H7 biofilm (Lee et al., 2011). Transcriptome analysis, in the second of these studies, showed that exposure to polyfloral, clover, and acacia honey significantly repressed the expression of curli, quorum sensing and virulence genes in *E. coli* O157:H7 (in *E. coli* phylogroup E). A separate study using qPCR of selected genes in exponential-growing *E. coli* Crooks ATCC 8739 (which like *E. coli* K-12 is in phylogroup A) exposed to clover, citrus, and marjoram honey gave similar results, showing particularly strong down-regulation of *tnaA*, the gene for indole biosynthesis; indole plays a signaling role in biofilm formation (Wasfi et al., 2016). SEM and FC showed that exposure of *E. coli* ATCC 14948 (also in phylogroup A) to natural multifloral unpasteurized honey disrupted the cell wall and caused filamentation, filament lysis, formation of spheroplasts and eventually cell lysis (Brudzynski and Sjaarda, 2014). These findings were recently confirmed in our study which also revealed that H_2_O_2_-producing honey compromises membrane polarity and integrity, an effect which is caused by the synergy of oxidative, acid and osmotic stress. We also identified significantly greater sensitivity to model honey (composed of sugars, gluconic acid, and H_2_O_2_) of catalase mutants (i.e., Δ*katE* and Δ*katG*) (Masoura et al., 2020). Transcriptome analysis of the effects of manuka honey on *Pseudomonas aeruginosa* showed synergies between different components, with effects on genes involved with the SOS response, oxidative damage, and maintenance of the proton motive force, among others (Bouzo et al., 2020).

Gene knockout screening is a useful tool to identify molecular targets of antimicrobials of known composition, but is challenging in the case of honey, due to its complexity in composition and the synergies between honey innate components that exert antibacterial activity. The use of a chemically defined model honey should allow to clarify the roles of the most abundant antimicrobial components and the interactions between them. We wished to understand the contribution of different genes to the overall ability of a given organism to survive a sub-lethal exposure to model honey, as this could aid in identifying which proteins and cellular pathways are the targets of the H_2_O_2_-producing honey. Here, we applied transposon directed insertion-site sequencing (TraDIS) to identify the genes with a role in resistance or susceptibility of *E. coli* K-12 MG1655 to stress caused by a model honey, as described in our previous work (Masoura et al., 2020). This model honey imposes the three main stressors (sugars, gluconic acid, and H_2_O_2_), involved in the Gox reaction and which are thought to be key components of the antibacterial activity of H_2_O_2_-producing honey. TraDIS involves the sequencing of high-density transposon libraries using next generation sequencing (NGS) to identify the location and relative frequencies of every insertion within a transposon library. This can then be used to determine the relative contribution to fitness of each gene before and after bacterial growth in the presence of specific stressors (Hayes, 2003; Langridge et al., 2009). The term “fitness” in this context is a measure of how well a cell competes with others to survive and reproduce in the presence of a given stress. Enrichment of strains containing insertions in that gene would indicate that deletion of that gene confers a fitness advantage to the population, while reduction would show that loss of function of this gene makes the strain less fit.

In this study, a transposon library was used to identify features important in the fitness of *E. coli* K-12 exposed to model honey. By this approach, we identified the genes in which transposon insertions caused a significant and time-dependent advantage (increase of fitness) or disadvantage (decrease of fitness) to *E. coli* post-exposure to model honey. Data were tested by construction and analysis of single knockout mutants. As a result of our analysis of these, we discuss pathways that may be of particular importance in understanding the impact of H_2_O_2_-producing honeys on *E. coli*.

## Materials and Methods

### Model Honey

The model honey was prepared by dissolving fructose (2.24 M), glucose (1.85 M), maltose (0.219 M) and sucrose (0.04 M) (all purchased from Sigma-Aldrich, United Kingdom) in deionized sterile water at 37°C as described previously (Bogdanov, 1997). Stock solutions of gluconic acid and H_2_O_2_ (Sigma-Aldrich, United Kingdom) were prepared in deionized sterile water and were added, at the appropriate concentrations, immediately before the start of the assay. The H_2_O_2_ stock contained stabilizer, which in theory could affect data interpretation, but we expect its effects if any to be small. The compositions of the model honey stocks (A and B) used in this study are presented in Table 1. The pH of model honeys (A; pH 4.1, B; pH 3.8) was measured using a Mettler Toledo pH meter (Mettler-Toledo Ltd., United Kingdom). The effects of model honeys were always assayed following dilution with an equal volume of bacterial cell culture.

**TABLE 1:** Composition of the model honeys.

Model honey	Sugars (w/v%)/gluconic acid (v/v %)/H_2_O_2_ (v/v %)	Fructose (M)	Glucose (M)	Maltose (M)	Sucrose (M)	Glu. acid (mM)	H_2_O_2_ (mM)
A	30%/0.14%/0.006%	0.8	0.66	0.07	0.014	9	2
B	30%/0.14%/0.009%	0.8	0.66	0.07	0.014	9	3

### Construction of Transposon Library

*E. coli* K-12 strain MG1655 was used for the construction of a transposon library. Briefly, 200 μL of *E. coli* K-12 MG1655 ultracompetent cells were transformed with 0.2 μL of EZ-Tn5™ < KAN-2 > Tnp Transposome™ (Kit; Lucigen, Cambridge, United Kingdom) and electroporated. Once electroporated, cells were recovered in SOC media (2% tryptone, 0.5% yeast extract, 10 mM NaCl, 2.5 mM KCl, 10 mM MgCl_2_, 10 mM MgSO_4_, and 20 mM glucose, all purchased from Sigma-Aldrich., United Kingdom) for 2 h at 37°C, plated onto LBA plates containing 30 μg/mL Kanamycin to achieve single colonies, and incubated overnight at 37°C. For a full library, the process was repeated 10 times with mutants being pooled and then stored using 10% glycerol at −80°C. Overall, a total of 450,581 unique insertion sites were identified corresponding to an average insertion every 10.3 bp.

### Single MG1655 Mutants

A total of 11 genes that were either significant enriched (*ompR*, *wecA*, *wecC*, *wecG*) or depleted (*fdoH*, *fdhD*, *moaA*, *moaB*, *moaC*, *gor*, and *prc*) in the TraDIS dataset (see Supplementary Table 3) were selected for validation experiments, and these genes were deleted from MG1655 using P1 transduction from the Keio library (Baba et al., 2006) into *E. coli* K-12 MG1655. All mutants were validated using PCR with appropriate gene-specific primers (Supplementary Table 4), as described previously in Thomason et al. (2007).

### Optimization Experiment for Antibacterial Assay

Overnight bacterial cultures were diluted in 5 mL Luria broth (LB) (10 g/L tryptone; 5 g/L yeast extract; 10 g/L NaCl; Sigma, United Kingdom) to a starting OD_600_ of 0.05 and incubated until the OD_600 nm_ reached 0.5 McFarland Standard (approx. 1.5 × 10^8^ CFU/mL). Before use, cells were pelleted (3900 g for 3 min in an Eppendorf centrifuge 5810), washed twice (pelleted, supernatant poured off, and cells resuspended with an equal volume of PBS), and resuspended again in PBS (BR0014, Oxoid Ltd., United Kingdom) to a final absorbance at 600 nm of 0.5. Resuspended cultures (100 μL) were mixed with equal volume (100 μL) of model honeys (Model A and Model B; Table 1) or PBS (as a negative control) and incubated at 22°C. Samples were taken at 30, 60, 90, and 120 min post-treatment and washed in PBS. After removing the PBS, each pellet was resuspended in 200 μL of LB, this inoculum was then transferred into 50 mL LB and incubated with shaking (150 rpm) at 37°C. Two hours post-incubation, each sample was washed twice and resuspended in equal volume of PBS. The antibacterial activity of the model honey was determined using total viable counts (TVC) and flow cytometry (FC).

### Flow Cytometry

A BD Accuri C6 flow cytometer (Becton Dickinson Biosciences, Oxford, United Kingdom) was used for FC analysis. For analysis of membrane permeability, samples were stained directly with 4 μg/mL Propidium iodide (PI) (Sigma, United Kingdom) and incubated at room temperature in the dark for 10 min. Untreated bacteria and bacteria treated with 3 M H_2_O_2_ for 30 min, served as “healthy” and “dead or membrane compromised” controls, respectively. For all assays, samples were excited using a 488 nm solid-state laser. PI fluorescence was detected using 670 LP filters. 25,000 data points were collected at a maximum rate of 2,500 events/sec and the data were analyzed using CFlow (BD) software.

### Treatment of the Transposon Library With Model Honey

For the transposon library, 10 μL of the library (∼2.4 × 10^9^ cells) were inoculated in 50 mL LB at a starting OD_600_ ∼0.05 and grown overnight at 37°C with shaking (150 rpm). 500 μL of the activated library was then inoculated into 50 mL of fresh LB and incubated until the OD_600_ reached 0.5 McFarland Standard. Before use, cells were pelleted (3,900 g for 3 min in an Eppendorf Centrifuge 5810), washed twice, resuspended in PBS (BR0014, Oxoid Ltd., United Kingdom) to a final absorbance of 1 OD_600_. 1 mL of the activated library was mixed with 1 mL model honey (model B) or 1 mL PBS (negative control). Samples were incubated for 30 or 90 min. Following this, samples were then subjected to 2 h outgrowth in fresh LB (as was described in optimization experiment). The biological replicate samples are referred in the text as TL30 (TL30_1, TL30_2), TL90 (TL90_1, TL90_2), and TL0 (TL0_1, TL0_2).

### DNA Extraction and Transposon Directed Insertion-Site Sequencing

Genomic DNA was isolated from all samples using a RTP^®^ Bacteria DNA Mini Kit according to the manufacturer’s specifications (Protocol 2: Isolation of DNA from bacteria pellets (1 × 10^9^ bacteria cells), STRATEC Molecular GmbH, Berlin). DNA was quantified using the Qubit™ dsDNA HS Assay kit (Invitrogen). 1 μg of gDNA was then fragmented using a bioruptor^®^ plus (Diagenode). NEB Next Ultra I kit (New England Biolabs, United Kingdom) was used to repair the ends of the fragmented DNA and to ligate an adaptor to the ends of the newly prepared DNA fragments. Following adaptor ligation, a PCR step was used to enrich the transposon junction fragments, using a custom forward primer annealed to the transposon end and a reverse primer annealed to the ligated adaptor. The second PCR step prepared DNA for sequencing through the addition of Illumina-specific flow cell adaptor sequences and custom inline index barcodes of variable length in the forward primers. The purpose of this was to increase indexing capacity while staggering the introduction of the transposon sequence, which increases base diversity during sequencing. Samples were sequenced using Illumina MiSeq 150 cycle v3 cartridges, aiming for an optimal cluster density of 900 clusters per mm^2^.

### Bioinformatics Analysis: Sequencing Analysis and Prediction of Gene Essentiality

Sequencing analysis was performed using custom scripts as described in Goodall et al. (2018). Read counts associated with transposon insertions were checked for any inline index barcode. Independently processed samples and short sequence reads were removed, and the data were then combined to increase the coverage resulting in 4,423,778 transposon-tagged reads which resulted in 450,581 unique insertion sites identified in the library. Insertion index scores were generated by dividing the number of unique insertions per gene by the gene length. Using this insertion index score, essential genes were identified using the procedure described in Goodall et al. (2018). This essential genome was then excluded from further analysis. For all remaining genes, relative fitness score (defined as log_2_-fold changes in read counts between the treated and control samples) were calculated using EdgeR within the ESSENTIALS package. Genes that showed a log_2_-fold change >1 or <−1 and an adjusted *p*-value of 0.005, after a Bonferroni correction was performed, were selected for further analysis (Zomer et al., 2012). Adjusted *p*-values and log_2_-fold changes for all genes for both T30 and T90 are shown in Supplementary Table 1.

### Gene Ontology Terms and Pathway Enrichment Analysis

The GO annotation of genes with a significant TraDIS score as defined above was done using the DAVID Bioinformatics Resources 6.7 and Gene Ontology Consortium Bioinformatics data bases (Ashburner et al., 2000). Any Biological Pathway that showed an enrichment for specific GO terms in the gene set with a *p*-value ≤ 0.005 was considered to be significantly enriched.

### Validation Experiments

Validation experiments involved testing the susceptibility of single knockout mutants to model honey and measuring relative selection rates against a *lacZ* derivative of MG1655. Susceptibility of the mutants was tested post-exposure to model honey B for 24 h as described above. Following overnight growth, strains were grown to the exponential phase (OD_600_ 0.5), washed twice in PBS and the OD_600_ was adjusted to 1. Each strain was combined with the *E. coli* Lac^–^ in a 1:1 ratio and was mixed with an equal volume (1 mL) of model honey B. Thirty (30) and ninety (90) min post-treatment with model honey, the cell pellet was harvested by centrifugation at 4,000 × *g* for 3 min and was resuspended in equal volume of PBS (2 mL). A 100 μL sample of the honey-treated samples (t30 and t90, respectively) and the control (cells kept in PBS) were plated on MacConkey Lactose agar and incubated overnight at 37°C, after which numbers of each strain were scored visually. As a measure of fitness, selection rate Lenski et al. (1991) was calculated for each experiment:


$$S\,e\,l\,e\,c\,t\,i\,o\,n\,r\,a\,t\,e\,\left(r\right)=\ln\left(\frac{R_{t}}{R_{0}}\right)-\ln\left(\frac{V_{t}}{V_{0}}\right)$$

Where r is defined as the relative competitive index, R (mutant) and V (WT) represent the two competing populations, 0 is the colony counts at timepoint zero (control) and t is the colony counts at timepoint 30 or 90 min, respectively.

## Results

### Optimization Step: Composition of Model Honey and Transposon Library Treatment

We used two model honeys with different concentrations of H_2_O_2_ to define one that caused logarithmic reduction without completely eradicating the bacterial population. These were model honey A and B, composed of 30% sugars, 8.6 mM gluconic acid, and 2 mM or 3 mM H_2_O_2_, respectively (Table 1). The bacterial viability was determined by colony counts and the phenotypic changes upon honey treatment were examined by flow cytometry (FC) using propidium iodide (PI), a DNA intercalating dye used as an indicator of cell membrane damage as it only enters cells with disrupted membranes (Nebe-von-Caron et al., 2000).

**FIGURE 1:**
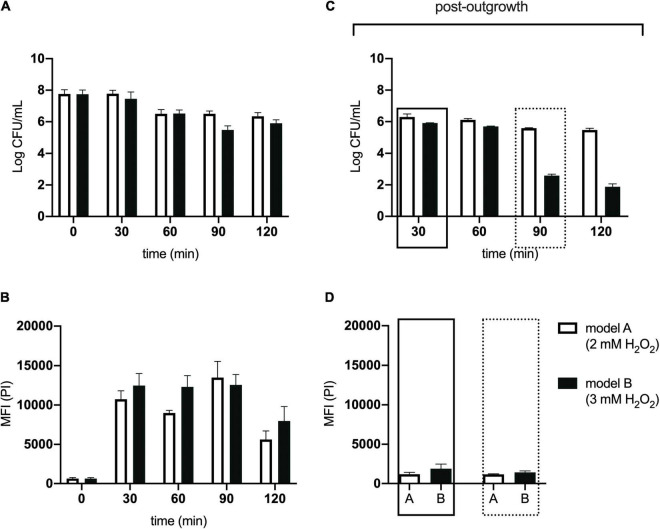
Antibacterial effect of model honeys on *E. coli*. Colony counts, showing the cell survival **(A)**, and mean fluorescence intensity (MFI) of PI, showing the extent of cell wall damage **(B)**, are plotted for 30, 60, 90, and 120 min post-exposure to honey model honey A (plain bars) and B (black bars). Colony counts **(C)** show the survival of the four treated populations after 2 h outgrowth in LB and the MFI of PI **(D)** of 30 min- and 90 min-treated population post- 2 h outgrowth in fresh LB. Error bars show standard deviations, (*n* = 3; biological replicates).

Within 90 min of treatment, model honey B (3 mM H_2_O_2_) caused almost 3 logs reduction while model honey A (2 mM H_2_O_2_) caused 1 log reduction, as assessed by viable counts (Figure 1A). FC showed that both model honeys affected cell membrane integrity within a few minutes of treatment. Cells exposed to model honey B showed consistently enhanced PI fluorescence, while in the case of cells exposed to model honey A, mean PI fluorescence intensity (MFI) also increased but fluctuated more during the 2 h of treatment (Figure 1B). This variation may be due to the bacterial catalase activity which degrades the H_2_O_2_ and to self-repair mechanisms that protect membrane integrity (Seaver and Imlay, 2001).

Following the treatment with model honeys, an outgrowth step was introduced as described by Phan et al. (2013). This step allowed the growth of all cells which had survived honey treatment and ensured that the cultures yielded enough genomic DNA (gDNA) for TraDIS sequencing. Figure 1C shows that the effect of both model honeys was time- and dose- dependent, with cfu dropping with longer exposure to honey (more slowly for treatment with model honey A). This may be due to a combination of irreversible cell injury/death and an extended lag phase caused by the stress (Hamill et al., 2020). Therefore, only intact and moderately injured cells are expected to grow in LB medium during the outgrowth step. FC showed that within the 2 h outgrowth, the surviving bacterial cells repaired their membranes, with the fluorescence of PI consequently decreasing compared to levels seen directly after treatment with model honey A and B (compare Figure 1D with Figure 1B).

### Exposure and Sequencing of Transposon Insertion Library to Model Honey

**FIGURE 2:**
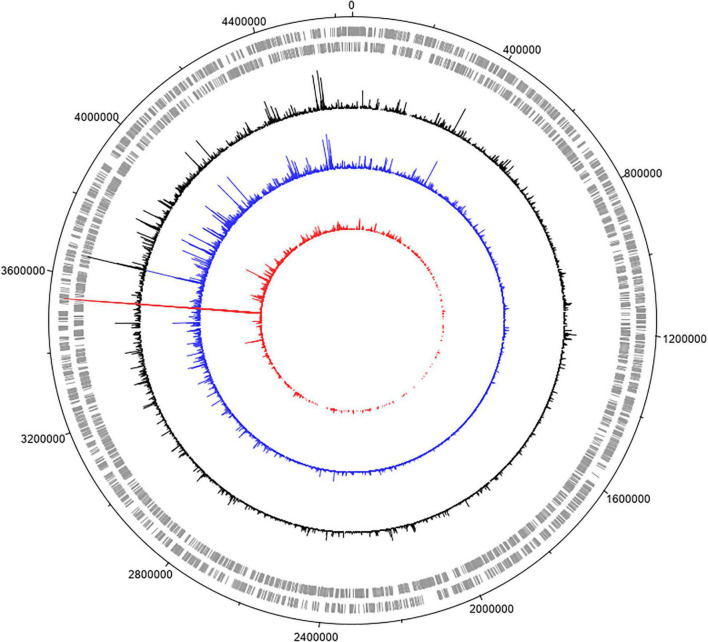
Genome-wide transposon insertion sites mapped to *E. coli* strain MG1655 in the TraDIS library. Frequency and location of transposon junction sequences from a mini-Tn*5* transposon library in strain MG1655, mapped to the MG1655 genome (CP009273.1). The outermost track marks the MG1655 genome in base pairs starting at the annotation origin. The next two inner tracks correspond to sense and antisense CDS, respectively. The innermost circles correspond to the frequency and location of transposons mapped to the MG1655 genome. The circles represent the initial MG1655 transposon library (black), and the library after 30 min (blue) and 90 min (red) exposure to model honey. The figure was created using DNAPlotter.

**FIGURE 3:**
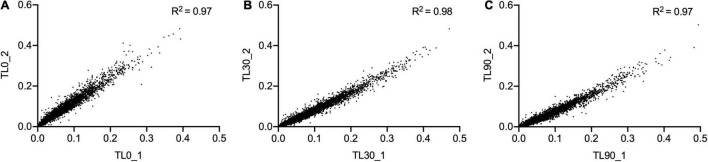
Replicates of TraDIS samples are highly correlated. Gene insertion index scores for all non-essential genes are shown for the two biological replicates of the control (input) transposon library **(A)**, and after selection in model honey for **(B)** 30 min (TL30_1, TL30_2) and **(C)** 90 min (TL90_1, TL90_2), followed by 2 h outgrowth in LB in both cases.

For these experiments we used a TraDIS library constructed in-house in the model *E. coli* K-12 strain MG1655. The distribution of insertion sites in this library covers the full genome with high frequency (Figure 2). Based on the results above, this library was exposed to model honey B (30% sugars, 8.6 mM gluconic acid, and 3 mM H_2_O_2_) for 30 and 90 min, followed in each case by 2 h outgrowth in LB. Given that *E. coli* quickly degrades H_2_O_2_, we hoped that the comparison of bacterial responses between these two time intervals (30 and 90 min) could give some insight into the synergy between honey components when H_2_O_2_ is more abundant (30 min) and when it is mostly degraded (90 min). The experiment was done twice, together with duplicate controls where the resuspended library was kept in phosphate-buffered saline (PBS) for equal times (30 and 90 min) without being treated with the model honey. The samples were labeled TL30_1, TL30_2, TL90_1, and TL90_2 for the 30- and 90-min time points of the two experiments, respectively, and two control samples were labeled TL0_1 and TL0_2. Reproducibility of the biological replicates was very high, as shown by a value of *R*^2^ (Pearson correlation) between 0.97 and 0.98 (Figure 3).

### Identification of Conditionally Essential Genes

**FIGURE 4:**
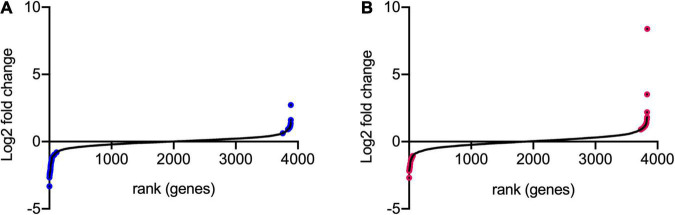
Log_2_FC of transposon insertion frequency for all non-essential genes. The log_2_FC for each non-essential gene was calculated for **(A)** 30 min stress/control and **(B)** 90 min stress/control. Log_2_FC values were then ranked from lowest to highest. Genes colored in blue and pink are those with logFC >1 or <–1 and with adjusted *p*-values < 0.005.

Transposon directed insertion-site sequencing data were analyzed by the ESSENTIALS pipeline (Zomer et al., 2012). The main output of ESSENTIALS is the log_2_-fold change (log_2_FC) of insertion counts between the treated and control samples, calculated by the EdgeR internal package (Robinson et al., 2010), and an adjusted *P*-value for each gene showing the level of significance of the measured log_2_FC. Figure 4 shows the log_2_FC for the whole bacterial genome following exposure to model honey for 30 and 90 min (TL30 and TL90), with genes ranked from smallest to largest log_2_FC. We took a log_2_FC of >1 or <−1 and an adjusted *P*-value of 0.005 to generate lists of genes where transposon insertion had a significant effect on strain fitness upon exposure to model honey.

Using this approach, we identified 42 (at TL30) and 41 (at TL90) genes where transposon insertion had a significant effect on strain fitness. Twenty-six of these were common between the two conditions (30 and 90 min) tested (listed in Table 2). A further 16 mutants showed a significant effect at 30 min but not at 90 min (Supplementary Table 2), and 15 had a significant effect at 90 min but not at 30 min (Supplementary Table 3). The identification of mutants with a role in fitness when exposed to model honey at 90 min only may be explained by their effect being time-dependent and relatively slow, but it is harder to explain why some mutants might have an effect at 30 min but not at 90 min. This point is considered further in the section “Discussion.”

**TABLE 2:** Genes that when mutated had a significant loss or gain of fitness at both 30 and 90 min following model honey challenge as identified by TraDIS.

Gene	Description	Operon structure	30’ (significant)		90’ (significant)	
			Log2 score	Adj. *p*-value	Log2 score	Adj. *p*-value
**Genes whose deletion caused loss of fitness on exposure to model honey**						
*prc*	Periplasmic tail-specific protease	*proQ-prc*	−3.323	1.13 × 10^–27^	−2.180	7.70 × 10^–13^
*moeB*	Molybdopterin-synthase adenylyltransferase	*moeAB*	−2.501	4.26 × 10^–23^	−1.646	0.0011
*moaA*	Molybdopterin biosynthesis: GTP 3′,8-cyclase	*moaABCDE*	−2.419	3.54 × 10^–15^	−1.288	0.0037
*fdoH*	Formate dehydrogenase O β subunit (FeS containing)	*fdoGHI-fdhE*	−2.393	6.72 × 10^–20^	−1.888	5.10 × 10^–09^
*selB*	Selenoprotein synthesis: Selenocysteine-specific elongation factor	*selAB*	−2.360	4.13 × 10^–24^	−1.901	8.01 × 10^–12^
*selA*	Selenoprotein synthesis: L-seryl-tRNA (Sec) selenium transferase	*selAB*	−2.274	5.02 × 10^–18^	−2.136	1.51 × 10^–09^
*fdoI*	Formate dehydrogenase O γ subunit (cytochrome *b*_556_)	*fdoGHI-fdhE*	−2.269	4.04 × 10^–11^	−1.889	4.39 × 10^–05^
*atpD*	ATP synthase β subunit	*atpABCDEFGH*	−2.257	3.99 × 10^–11^	−1.837	0.0003
*yohD*	Inner membrane protein	*yohD*	−2.245	2.25 × 10^–07^	−1.957	0.0017
*fdhE*	Formate dehydrogenase formation protein	*fdoGHI-fdhE*	−2.128	1.16 × 10^–11^	−2.047	2.15 × 10^–06^
*fdhD*	Sulphurtransferase for molybdenum cofactor sulphuration	*fdhD*	−2.103	4.48 × 10^–12^	−1.869	7.38 × 10^–06^
*fdoG*	Formate dehydrogenase O α subunit (MGD-containing, selenoprotein)	*fdoGHI-fdhE*	−2.070	1.26 × 10^–25^	−2.035	1.85 × 10^–15^
*selD*	Selenoprotein synthesis: Selenide, water dikinase	*selD-topB*	−1.931	1.45 × 10^–06^	−2.685	5.57 × 10^–09^
*moeA*	MoCo biosynthesis: Molybdopterin molybdenumtransferase	*moeAB*	−1.467	6.25 × 10^–09^	−1.200	0.0009
*glnG*	DNA-binding transcriptional regulator NtrC	*glnALG*	−1.215	8.43 × 10^–05^	−1.391	3.41 × 10^–05^
*fetA*	Iron export ABC transporter *fetAB*	*fetAB*	−1.149	0.0002	−1.172	5.61 × 10^–05^
*fetB*	Iron export ABC transporter *fetAB*	*fetAB*	−0.961	0.0026	−1.061	0.0041
**Genes whose deletion caused gain of fitness on exposure to model honey**						
*rep*	ATP-dependent DNA helicase	*rep*	0.913	0.0002	1.263	6.97 × 10^–10^
*acnB*	Aconitate hydratase B	*acnB*	0.940	0.0023	1.182	1.39 × 10^–05^
*wecC (rffD)*	ECA biosynthesis: UDP-N-acetyl-D-mannosamine dehydrogenase	*wecA-wzzE-wecBC-rffGHC-wecE-wzxE-wecF-wxyE-wecG*	0.947	5.14 × 10^–05^	1.212	6.58 × 10^–06^
*envZ*	EnvZ/OmrR Two-component signal transduction system	*ompR-envZ*	1.030	7.95 × 10^–06^	3.522	1.70 × 10^–44^
*wecB (rffE)*	ECA biosynthesis: UDP-N-acetylglucosamine 2-epimerase	*wecA-wzzE-wecBC-rffGHC-wecE-wzxE-wecF-wxyE-wecG*	1.035	2.23 × 10^–12^	1.335	1.11 × 10^–11^
*wecG (rffM)*	ECA biosynthesis: UDP-N-acetyl-D-mannosaminuronic acid transferase	*wecA-wzzE-wecBC-rffGHC-wecE-wzxE-wecF-wxyE-wecG*	1.044	1.59 × 10^–05^	1.203	6.74 × 10^–05^
*wecA (rfe)*	ECA biosynthesis: UDP-N-acetylglucosamine—undecaprenyl-phosphate N-acetylglucosaminephosphotransferase	*wecA-wzzE-wecBC-rffGHC-wecE-wzxE-wecF-wxyE-wecG*	1.045	8.17 × 10^–07^	1.092	0.0002
*tolA*	Tol-Pal system protein	*ybgC-tolQRA*	1.148	0.0031	1.174	0.0009
*ompR*	EnvZ/OmrR Two-component signal transduction system	*ompR-envZ*	1.613	6.99 × 10^–12^	8.392	5.06 × 10^–134^

*The genes are ranked according to the log_2_FC. Operon structures are from Ecocyc.org.*

### Gene Ontology Analysis of Transposon Directed Insertion-Site Sequencing Data

**FIGURE 5:**
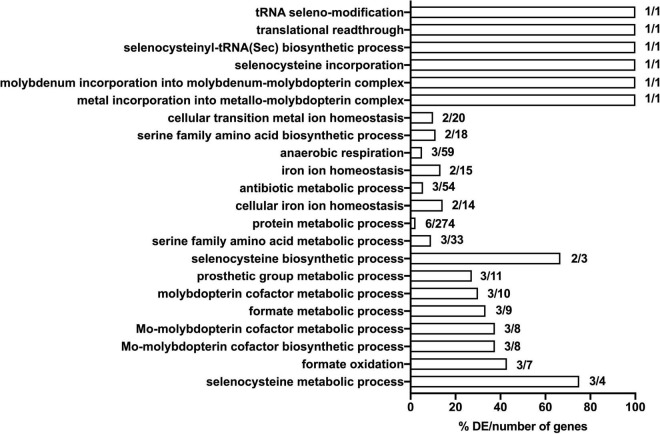
Significantly enriched gene ontology (GO) terms for the seventeen mutants that show significant loss of fitness following 30- and 90-min exposure to model honey. Genes included in the analysis were these whose corresponding mutants had a logFC >1 or <–1 with adjusted *p*-values < 0.005. Comparisons were made using the number of genes associated with each term in the gene set versus the total number of genes associated with each term in the (*E. coli* MG1655) genome. The respective percentage is given in *x* axis.

In order to gain preliminary insight into the possible function of genes identified in the TraDIS analysis, we undertook a basic gene ontology analysis of selected TraDIS data. Specifically, we analyzed genes where insertions caused a significant loss of fitness at both the 30- and 90-min time points (with significance defined as above), looking at the biological processes that were enriched in the genes represented in the lists. The annotation analysis was conducted in terms of their biological pathway. The results (shown in Figure 5) suggests that selenocysteine, Mo cofactor biosynthesis and the activity of formate dehydrogenases (FDHs) were the most significantly affected pathways upon exposure to model honey. The roles of these genes, plus other genes in the significant lists, and the possible explanations of their effects on fitness in honey treated cells, are considered in the section “Discussion.”

### Validation of Transposon Directed Insertion-Site Sequencing Data

**FIGURE 6:**
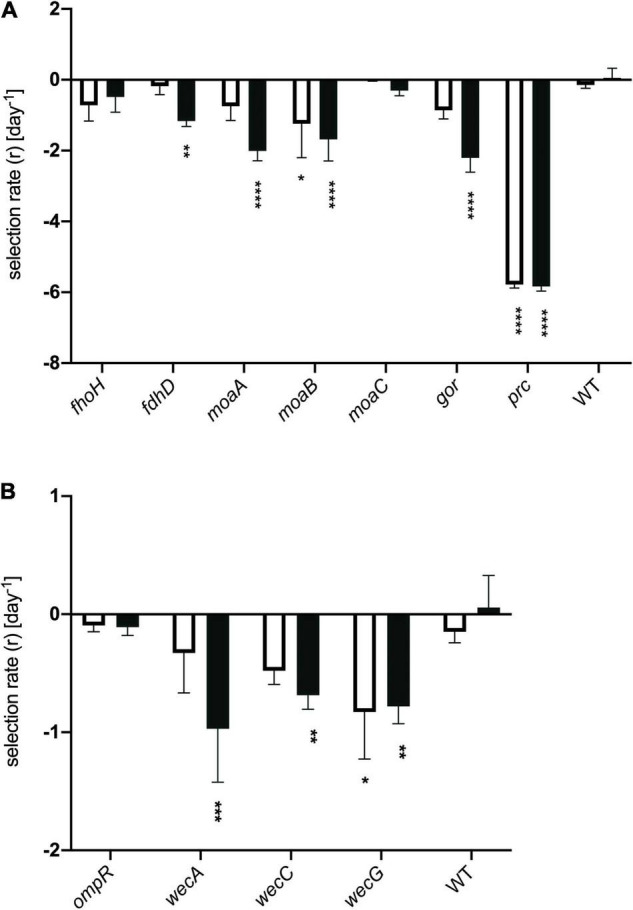
Selection rates for strains deleted for individual genes. Genes classified in the TraDIS dataset as having **(A)** negative fitness or **(B)** positive fitness when containing transposon insertions. Strains were mixed with represented their wild type parent (*E. coli* MG1655, marked with a *lacZ* mutation) in model honey for 30 min (plain bar) or 90 min (black bar), before plating on MacConkey lactose to determine relative survival rates. The WT control shows the data for MG1655 *lacZ* mixed with MG1655 LacZ^+^. Data were analyzed using two-way ANOVA: asterisks show significance levels of Sidak’s multiple comparisons test (****p ≤ 0.0001, ***p ≤ 0.0005, **p ≤ 0.005, *p ≤ 0.05); all bars without asterisks are not significant (*p* > 0.05). Error bars represent the average ± SD (*n* = 3; independent biological replicates).

Having identified genes whose inactivation has an impact on the fitness of honey-treated cells, we attempted to validate a representative selection of these by creating deletions of the appropriate genes and testing their effects on bacterial fitness relative to wild type when exposed to model honey B, as used for the TraDIS experiment. As described in section “Materials and Methods,” we used assays that measured relative rates of killing in mixtures of the wild type strain with individual knockout strains (having marked the wild type with a *lacZ* mutation for ease of screening on MacConkey-lactose plates; control experiments confirmed this mutation had no effect on strain fitness). We also directly measured the sensitivity of the individual mutants to model honey B. Deletions of eleven of the genes were engineered by P1 transduction from the Keio library (Baba et al., 2006), selecting for kanamycin resistance, as described in Materials and Methods. All deletions were verified using PCR. Figure 6 shows the fitness of these eleven deletion mutants relative to the wild type strain with the *lacZ* marker gene. The relative rate of killing is equivalent to the selection rate and was calculated as described in Lenski et al. (1991).

In the case of genes where transposon insertion caused a significant decrease in fitness in the TraDIS experiment, loss of strain fitness was also generally seen when these genes were deleted (Figure 6A). Deletions of *moaA, moaB*, *fdhD, gor* and *prc* showed agreement with the TraDIS data, showing significantly reduced fitness relative to the wild-type after 90 min of exposure to honey. The *prc* mutant, in particular, was very rapidly eliminated in these experiments, with zero viable colonies being seen after 30 and 90 min of exposure to honey; it also showed the largest log_2_-fold decrease in the TraDIS data. Deletion of other genes also showed an impact on fitness though not always with an identical pattern to the TraDIS data. Deletion of *fdhD* was significant at both times in TraDIS, but only at 90 min in the competition experiment. Interestingly, deletion of *gor* was only just significant at 30 min and not at all at 90 min in the TraDIS experiment but had a significant impact on fitness only at the 90 min time point in the competition experiment. Conversely, *fdoH* was clearly significant at both time points in the TraDIS experiment, but only a slight and non-significant decline in fitness was seen in the competition experiment when this gene was deleted. Also, TraDIS showed that the loss of function both *moaA* and *moaB* was more significant at 30 min compared to 90 min, while the competition experiments showed the alternative with both mutants having a higher loss of fitness at 90 min compared with 30 min.

**FIGURE 7:**
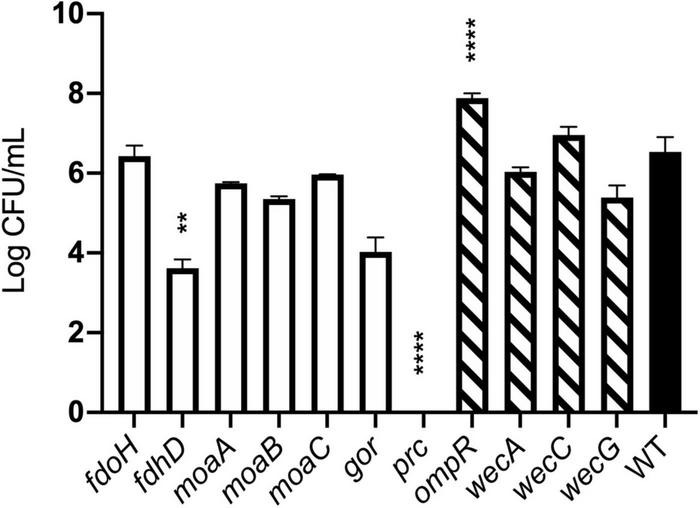
Effect of model honey B on strains deleted for individual genes. Strains containing deletions of genes previously identified as “loss of fitness” (plain bars) and “gain of fitness” (hatched bars) were treated with model honey B for 24 h before plating to measure survival. ****p ≤ 0.0001, **p ≤ 0.005. The graph shows average ± standard deviation (*n* = 3; biological replicates).

The agreement between competition and TraDIS experiments was less clear for genes where transposon insertion caused a gain of fitness in the TraDIS. Transposon inserts in *ompR* showed the most significant increase in relative fitness in the TraDIS experiments, particularly at 90 min. However, in the competition experiments, the *ompR* deletion strain showed a slight but non-significant decline in fitness relative to wild-type (Figure 6B). Moreover, inserts in the *wecA*, *wecB*, *wecC*, and *wecG* genes all caused slightly increased fitness in the TraDIS experiments, but deletion of these genes caused significant loss of fitness relative to wild type in the competition experiments. Because of the discrepancies seen with some of the mutants, a separate measurement was done of the susceptibility of each of the mutants to 24 h exposure to model honey (B), as an independent way to evaluate the impact of loss of function of these genes on strain fitness. To define the severity of a phenotype, thresholds were set to cluster the mutants into three groups; (a) “weak” for mutants showing less than 2 logs reduction, (b) “intermediate” for those showing 3–4 logs reduction and (c) “strong” those with higher than 5 logs reduction. Figure 7 shows that Δ*fdhD*, Δ*gor*, and Δ*prc* were the most susceptible strains to model honey. In agreement with both TraDIS and the competition assay, Δ*prc* demonstrated a strong loss of fitness phenotype followed by Δ*gor* and Δ*fdhD* which both had intermediate phenotypes. The rest of the mutants (Δ*moaA*, Δ*moaB*, Δ*moaC*, and Δf*doH*) had an intermediate phenotype apart from Δ*ompR* and Δ*wecC*, both of which were more resistant than the wild type, although this was only statistically significant in the case of the Δ*ompR* strain.

Overall, most of the phenotypes observed in these experiments agreed with TraDIS analysis. Thus, *prc*, *gor*, and *fdhD* are the genes which under the conditions of our experiment are most important to enable the bacteria to resist the effects of the model honey, while loss of *ompR* led to better survival than the wild type in the presence of model honey.

## Discussion

**FIGURE 8:**
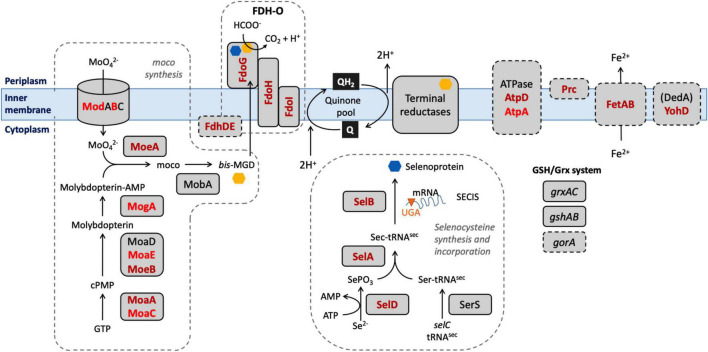
Selected biological pathways where the “loss of fitness” genes are involved. Dashed boxes include biological pathways and genes which were shown to be significantly affected after the exposure to honey. Mo cofactor and selenocysteine, being incorporated into the 3 FDHs after the biosynthesis, are denoted by yellow and blue polygons, respectively. Genes found in both lists are shown in dark red; genes found only in the TL30 list are shown in light red.

Our aim in this study was to identify genes in *E. coli* which, when their function was lost, caused a significant change in the ability of the organism to survive exposure to a model honey. Our choice of model honey was based on previous work showing that dilution of honey to 30–50% of its initial concentration led to H_2_O_2_ concentrations between 0.04 and 4 mM, and gluconic acid concentrations between 8.6 and 60 mM (Manyi-Loh et al., 2011; Bucekova et al., 2018). Identification of these genes should provide insights into the role of particular pathways in surviving the stress caused by the model honey, which in turn can give further information about the mechanisms by which honey causes cell damage and death (Figure 8). *E. coli* K-12 MG1655 was used for the experiments described here and in our earlier study because of the large amount of available prior knowledge of gene function in this organism; subsequent work on clinical isolates and other bacterial species will be needed to see the extent to which our findings can be generalized. Here, we consider those genes that caused a loss of fitness when disrupted, where data from single gene knockouts agreed with data from the TraDIS experiments. We then briefly consider the smaller number of genes where disruption caused a gain of fitness relative to the wild type strain, although with these genes, data from the two assay methods were not always consistent.

Mutation of seventeen genes caused a significant loss of fitness at both time points in the treatment with model honey (*fetB* is included although it is fractionally below the cut-off for impact on fitness at 30 min). Of these, eleven are involved directly or indirectly in formate oxidation, encoding formate dehydrogenase O (FDH-O; *fdoGHI*), or proteins involved in providing FDH-O with its molybdenum-containing cofactor (molybdenumtransferases; *moeAB*, molybdenum cofactor; *moaA*, and the *fdhED* genes), or proteins involved in selenocysteine biosynthesis (*selABD*). Selenocysteine is an amino acid present in the three formate dehydrogenases. GO term analysis of this list confirms that pathways affecting selenocysteine biosynthesis, Mo cofactor biosynthesis and the activity of formate dehydrogenases (FDHs) were the most significantly enriched pathways where mutation caused loss of fitness on exposure to model honey.

In *E. coli*, FDH-O is one of three formate dehydrogenases (FDH-N, FDH-O, and FDH-H) that have a role in formate oxidation. All contain selenocysteine (sec), moco (molybdenum cofactor), iron-sulfur (4Fe-4S) clusters, and require the FdhE and (with the exception of FDH-N) FdhD proteins for maturation (Lüke et al., 2008). The products of four *sel* genes (*selABCD*) have been identified as prerequisites for the biosynthesis and incorporation of sec into selenoproteins. Strains carrying mutations in *selD*, *selA*, and *selB* all significantly lost fitness upon honey treatment which suggests that one or more selenoproteins are important for bacterial survival under the tested conditions, the most likely candidate being FDH-O itself as it is the only selenoprotein also encoded by members of the list of significant genes. Molybdenum, an essential transition metal in biological systems, is involved in redox activities and has a key role in PMF generation (Leimkühler, 2014). Most of the terminal reductases that take electrons from FDH-O are also molybdoproteins (NarGHI, NarZYV, TorAC, DmsABC). The *moa* and *moe* operons encode proteins that are directly involved in the biosynthesis of moco (Coughlan, 1983). The deletion of genes involved in molybdoprotein synthesis increased the susceptibility of *E. coli* toward HAP (6-N-hydroxylaminopurine) and chlorate due to the reduced reductase activity which caused an imbalance in the PMF (Kozmin and Schaaper, 2013) and destruction of membrane polarity and integrity (Riondet et al., 1999). Again, their appearance in the list of significant genes is likely be due to their role in FDH-O function.

During formate oxidation, FDH-O donates electrons to the quinone pool and can contribute to the proton motive force (PMF) in a redox loop with nitrate reduction under anaerobic conditions (Abaibou et al., 1995). The role of FDH-O in anaerobic growth is well studied, but its role in aerobic growth is less well understood, despite the fact that it is produced constitutively under aerobic conditions. Recent observations suggest that it has a role in survival of stress caused by ROS. Over-production of the electron transfer sub-units of FDH-O (*fdoH* and *fdoI*) causes an increase in resistance in stationary phase to menadione, a redox cycling agent that generates H_2_O_2_ and superoxide (Iwadate et al., 2017). This activity does not require the formate dehydrogenase activity *per se*, so its connection with our finding that mutation of gene encoding the α sub-unit of FDH-O (*fdoG*) also causes sensitivity to model honey is not clear. A study using Tnseq (closely related to TraDIS) showed that strains carrying mutations in seven of the genes that we detected (*fdhE*, *fdhD*, *fdoH*, *fdoG*, *selA*, *selB*, and *moaA*) showed enhanced resistance to killing by mammalian peptidoglycan recognition proteins (PGRPs) (Kashyap et al., 2020). The authors propose this may arise because in wild type cells treated with PGRP, FDH-O is activated, leading to an increased flow of electrons into the respiratory chain and generation of damaging hydrogen peroxide by cytochrome *bd*, which is functioning incorrectly due to the action of the PGRPs. This hypothesis in turn suggests a possible model for this part of our current findings. Cytochrome *bd* is itself an effective quinol peroxidase, reducing hydrogen peroxide to water and using ubiquinol as an electron donor, and has been proposed to be an important scavenger of exogenous hydrogen peroxide (Al-Attar et al., 2016). Ubiquinol can be generated by FDH-O catalyzed oxidation of formate (Hartwig et al., 2015). Therefore, one of the routes that *E. coli* can potentially use to defend itself against the hydrogen peroxide generated in model honey is via the ubiquinol-dependent peroxidase activity of cytochrome *bd*, and this would be reduced if FDH-O were absent or inactive, consistent with our TraDIS data. The genes for the other membrane located formate dehydrogenases (FDH-N) showed no evidence of loss of fitness when mutated in the TraDIS experiment, consistent with the fact that FDH-O is expressed aerobically but FDH-N is not (Abaibou et al., 1995). We note that cytochrome *bd* is itself essential under the conditions of our experiment, so would not generate a TraDIS signal.

A plausible hypothesis for the roles of the *fetAB* genes, which were significant at both time points, is also suggested by their known functions. FetAB is a membrane transporter that exports Fe^2+^ from cells, and deletion of *fetA* or *fetB* results in increased sensitivity to H_2_O_2_, presumably because increased intracellular Fe^2+^ favors the Fenton reaction that leads to disproportionation of hydrogen peroxide and the production of damaging ROS (Nicolaou et al., 2013). The fact that mutation of these genes also causes increased sensitivity to model honey in our experiments suggests that the production of ROS by the Fenton reaction is a key factor in the antibacterial effect of honey.

Roles for the remaining genes in the list based on their known functions are more speculative. The *prc* gene is of particular interest, as both TraDIS and analysis of the Δ*prc* strain showed strains lacking Prc were extremely susceptible to treatment with honey. Prc is a periplasmic protease which cleaves the C-termini of several proteins, and its loss causes a range of effects including increased outer membrane permeability, periplasmic protein leakage, and sensitivity to several antibiotics (Hara et al., 1991; Seoane et al., 1992; Deng et al., 2014). In extraintestinal pathogenic *E. coli*, *prc* mutants are attenuated in a mouse model of urinary tract infections (Huang et al., 2020), where deletion of *prc* has been implicated in perturbation of the cell envelope and induction of the Rcs and σ^E^ regulons. These examples show that Prc has an important extra-cytoplasmic role, but the pleiotropic nature of the mutant makes it difficult to propose a specific mechanism for its role in the context of honey treatment. The strength of the phenotype suggests this would repay further study. The *yohD* gene has not been much studied but is a member of the DedA family of proteins that have a role in membrane homeostasis, with mutants showing phenotypes such as cell division defects, altered membrane lipid composition, loss of PMF and increased envelop-related stress responses. For this reason, DedA proteins have been studied as a potential drug target (Doerrler et al., 2013). *E. coli* has eight DedA proteins (YghB, YqjA, YabI, DedA, YdjX, YdjZ, YqaA, and YohD) but other than YohD, none of these showed any significant change in the TraDIS data, and further study of the role of this particular DedA protein in protecting against honey would therefore be of interest. The *atpD* gene encodes a sub-unit of the F_1_ component of the F_0_F_1_-ATPase; the other two non-essential sub-units (*atpA* and *atpG*) also showed loss of fitness though not all below the stringent *p* < 0.005 cut-off we have applied here. Loss of function of this complex will both reduce ATP levels in cells, with a negative effect on ATP-requiring processes, and also may prevent the ATP-driven efflux of protons which is used in some organisms, possibly including *E. coli*, as a defense against acidification (Sun et al., 2012). As model honey (which has a pH of 3.8) contains gluconic acid which has a pKa of 3.86, 50% of the gluconic acid will be in the non-ionized state and could easily cross the bacterial inner membrane, where it will ionize in the bacterial cytoplasm, potentially causing acid stress, although we did not see evidence for loss of fitness caused by mutations in any of the known acid response genes. The *glnG* gene (also known as *ntrC*) is part of the NtrBC two component system which responds to nitrogen limitation in *E. coli* (Jiang and Ninfa, 1999). Intriguingly, *glnL* (*ntrB*) also caused loss of fitness when mutated, as did *rpoN*, the gene for the sigma-54 sub-unit of RNA polymerase, which is needed for the expression of many NtrC-regulated genes, although neither of these genes were significant enough to be included in our lists. For now, the reason why these genes show a TraDIS signal remains unclear, as *E. coli* at exponential phase in LB is not expected to be nitrogen-limited.

Of the mutations causing significant loss of fitness in the TraDIS data at 30 min only, several are in genes involved in moco biosynthesis (*moaC*, *moaE*, *modB*, and *mogA*) and also show loss of fitness at 90 min, though not at significant levels. This may reflect higher variability in the TraDIS values for these genes in the 90 min dataset and is consistent with the central importance of the FDH-O-ubiquinone-cytochrome *bd* pathway proposed above. The *atpA* gene, another non-essential component of the F1 ATPase complex, is also in this category, consistent with the proposed role of this complex in reducing intracellular proton concentration. The *gntR* gene also shows significant loss of fitness at 30 min and evidence of loss of fitness at 90 min. The GntR protein regulates both gluconate metabolism and the import of gluconate, and the negative fitness seen with loss of this gene may be caused by depression of the gluconate I and II import systems which would cause even more stress due to high cytoplasmic gluconate concentration (Rodionov et al., 2000). Of the remaining genes in the list, polyphosphate kinase (*ppk*) has been identified as having a role in resistance to H_2_O_2_ and heat shock (Crooke et al., 1994). The *Salmonella* Typhimurium *mrp* homolog, *apbC*, is thought to have a role in FeS center metabolism (Skovran et al., 2004) so is potentially involved in maintenance of redox enzymes. Glutathione reductase (*gor*) reduces oxidized glutathione (Lu and Holmgren, 2014) thus can be implicated in repair of oxygen-mediated damage. We suggest that all these genes may represent response mechanisms especially important in the short term after exposure to model honey; further studies on individual mutants will be needed to test this.

Only four genes showed a significant loss of fitness in the TraDIS data at 90 min but not 30, and these were only marginally significant compared to most of the genes that lost fitness at both time points, showing that most of the fitness effects detected by TraDIS occur during the early stages of exposure to model honey. Gluconate is degraded in *E. coli* by conversion into 6-phosphonogluconate which is then fed into the pentose phosphate pathway by the activity of 6-phosphogluconate dehydrogenase, encoded by *gnd*, so the deleterious effect of mutations in this gene may be caused by limitation the cell’s ability to metabolize the gluconate component of model honey. The reasons for the other phenotypes for mutants in this list are not clear.

There were some surprises at genes that did not meet our significance criteria. For example, neither *katE* nor *katG*, two genes that we had previously shown to cause increased sensitivity to model honey appeared in the significant gene lists, although *katE* only narrowly missed inclusion in the 90-min list. This may reflect a difference in the assay methods: in a TraDIS experiment, the overwhelming majority of strains in the population will be wild type for these two genes, so the rare loss of function mutants may benefit from the activity of the majority of strains carrying these wild type genes in the population. We also looked at the fitness of *tnaA* mutants, as this gene has been reported to be very strongly down-regulated on exposure to honey (Lee et al., 2011; Wasfi et al., 2016), but there was no fitness defect associated with these mutants under our conditions. The loss of OxyR, the regulator which controls the expression of genes to combat oxidative stress, had a small deleterious fitness effect at both time points which again was not significant enough to be included in our list, consistent with the view that the effects of hydrogen peroxide are not the only cause of the impact of model honey.

Finally, we turn to the phenomenon of genes whose loss of function caused gain of fitness in the TraDIS experiments. The data here are less clear as the measurements of relative killing rates using the mixed populations, and sensitivity of individual mutants, did not always correlate with the TraDIS data, so these may be phenotypes which are only observed when the mutants are present as a very small proportion of the population. We are currently investigating this in more detail.

The appearance of three genes from the *wec* operon in this list, together with *tolA*, was particularly intriguing. This operon (*wecABCG*) encodes some of the proteins required for synthesis and translocation of the Enterobacterial Common Antigen (ECA) (Danese et al., 1998). ECA mutants are viable but have an elongated and swollen cell morphology, suggestive of an altered cell envelope. In *E. coli*, mutants of the ECA synthesis pathway have an activated Rcs phosphorelay system that is required for inducible acid resistance in *E. coli* (Johnson et al., 2011; Castelli and Véscovi, 2011). Rcs is also activated by mutants of the Tol-Pal system which has a role in maintaining lipid homeostasis in the outer membrane (Clavel et al., 1996; Shrivastava et al., 2017). It has been shown that Tol mutants show enhanced sensitivity to vancomycin, and this is rescued by mutants in the *wec* operon (Jiang et al., 2020). Thus, the fact that both *tolA* and *wec* mutants show enhanced resistance to model honey could be directly related to the defect in ECA biosynthesis or to the induction of the Rcs phosphorelay, and this will repay further investigation.

The most striking example of genes which, when mutated, caused increased fitness in the presence of model honey were *ompR* and *envZ*. Together, these encode a two-component system which has important roles in response to several stresses, including osmotic stress and low pH. Generally, this is due to the phosphorylated form of OmpR which is an activator, but non-canonical behavior that does not require phosphorylation is also important (Kenney and Anand, 2020). Why should loss of function of these genes lead to a significant enhancement in resistance to model honey, particularly in TraDIS experiments but much less so when looking at single mutants? The EnvZ-OmpR two-component system regulates bacterial response to acid and osmotic stress, and its activity affects the acidification of the cytoplasm via repression of the *cadA*, *cadBC*, and *speF* genes (Stincone et al., 2011; Quinn et al., 2014; Chakraborty et al., 2017; Chakraborty and Kenney, 2018). For instance, on exposure to pH 5.6, the *E. coli* cytoplasm was shown to acidify from pH 7.13 to 6.55 in an OmpR-dependent manner, due to repression of the *cadA* and *cadBC* genes by OmpR. High osmolarity was also shown to result in acidification of the cytoplasm (to pH 6.75), but by a different pathway: repression *speF*, encoding ornithine decarboxylase, by OmpR. OmpR requires contact with EnvZ, but not phosphorylation, for this regulation to occur. An *ompR* mutant retained a neutral cytoplasmic pH in response to both pH 5.6 and osmotic stresses. Although the decrease in cytoplasmic pH seen in acid or osmotic stress by Chakraborty et al. is likely to be advantageous in surviving a single stress, it may be that the combination of stresses delivered by model honey means that maintaining a neutral cytoplasmic pH (as would occur in an *ompR* or *envZ* mutant) improves resistance to one or more components in model honey. This would explain the apparent fitness advantage of mutants in these genes that was seen in the TraDIS data. However, other effects such as the impact of *envZ* and *ompR* mutants on the expression of OM porins, that are needed for the passive diffusion of small hydrophilic molecules such as β-lactams and fluoroquinolones, (Ceccarelli and Ruggerone, 2008), cannot be ruled out.

Some genes showed increased fitness in the TraDIS data at 30 min only or 90 min only, though many of these effects were relatively small. At 30 min, *ymjC* had the strongest apparent advantage; however, no reads corresponding to this gene were found in the 90 min dataset. The *ymjC* gene is small (138 base pairs) and hence has very few inserts, which makes it more susceptible to variation due to stochastic effects alone in a TraDIS population, so this data must be interpreted cautiously. Similarly, knockout of eleven genes significantly increased fitness at 90 min post-challenge but not at 30 min (Supplementary Table 2), though some of these levels were again marginal. Most of these genes have previously been linked to stress responses and four are involved or implicated in envelope integrity. The first of these, the small RNA *mgrR*, regulates lipopolysaccharide remodeling and plays a role in ROS response (Klein and Raina, 2015). However, TraDIS data for this small gene (98 base pairs) has to be interpreted with caution for the same reasons given for *ymjC* above. Of the other genes, PlsX is involved in phospholipid biosynthesis (overexpression of *plsX* resulted in increased tolerance to the solvent styrene (Machas et al., 2021), suggesting a strengthening of the membrane against some stresses); the porin OmpC has a role in response to envelope stress such as salt, ethanol and SDS (Choi and Lee, 2019); and finally MepS (Spr) remodels the peptidoglycan; a *mepS* deletion prevents growth at high temperatures (42°C) on nutrient agar (Singh et al., 2015). The reasons why mutations in these genes appear to cause improved resistance to the effects of 90 min exposure to model honey are not clear, although it is likely to be related to the importance of the bacterial envelope. Compromising envelope integrity is likely to be associated with sensitivity to components in model honey, consistent with our results above and in Masoura et al., 2020, but some alterations to the envelope may improve resistance.

In addition, three regulators caused fitness improvement at 90 min when mutated. ComR regulates copper acquisition (Rademacher and Masepohl, 2012). ComR regulates expression of *bhsA*, which is also upregulated in the presence of styrene (Machas et al., 2021). Knockout of *qseC* (encoding a sensor kinase involved in motility regulation) increases sensitivity to several cations (Zhou et al., 2003). The oxygen-sensing diguanylate kinase DosC regulates biofilm formation; its expression is upregulated in stationary phase in an RpoS-dependent manner (Weber et al., 2006). Finally, three poorly characterized genes are implicated in resistance to diverse stressors: *yciF* is upregulated under osmotic stress (Weber et al., 2006); overexpression of *yejG* increases resistance to some antibiotics (Soo et al., 2011) and *yejG* is upregulated in response to citral (Chueca et al., 2017); *ydcT*, encoding a putative ABC transporter, is implicated in triclosan resistance (Zheng et al., 2001). Thus, in all cases these genes are involved in different aspects of stress resistance, but we currently do not have simple explanations for why mutations in them can enhance fitness under our experimental conditions. Overall, these data suggest that the pathways that allow adaptation to model honey overlap many other stress responses in *E. coli*.

We have thus used a range of physiological and molecular approaches to characterize the mechanism of action of a defined model honey. Even though the honey has only three chemical components, its deleterious effects on *E. coli* appears to be mediated through multiple pathways and mechanisms, consistent with synergies between these components. We specifically propose the involvement of cytochrome *bd* in detoxification of the hydrogen peroxide which is produced when the honey is activated, but many other pathways are also clearly involved. Our model honey represents a baseline for understanding the anti-microbial mechanism in H_2_O_2_-producing honeys. The method we describe here can be extended by adding other important components (such as polyphenols) to model honey and looking at the additional impacts of this, and by using clinically important isolates as target organisms in such experiments. Ultimately, this research should provide understanding that may improve the modification of natural honey into bio-engineered formulations with medicinal use.

## Data Availability Statement

The original contributions presented in the study are included in the article/Supplementary Material, further inquiries can be directed to the corresponding author/s.

## Author Contributions

MM, TO, PL, and KG conceived and designed the experiments. MM executed the experiments and together with MTM performed the TraDIS experiment. MTM analyzed the TraDIS data and Gene Ontology. MM wrote the manuscript. TO, MTM, PL, and KG critically reviewed the manuscript. PL acted as the primary supervisor of this work. All authors have read and approved the manuscript.

## Conflict of Interest

The authors declare that the research was conducted in the absence of any commercial or financial relationships that could be construed as a potential conflict of interest.

## Publisher’s Note

All claims expressed in this article are solely those of the authors and do not necessarily represent those of their affiliated organizations, or those of the publisher, the editors and the reviewers. Any product that may be evaluated in this article, or claim that may be made by its manufacturer, is not guaranteed or endorsed by the publisher.
