# Supplementary material for: Use of Transposon Directed Insertion-Site Sequencing to Probe the Antibacterial Mechanism of a Model Honey on E. coli K-12
Source: Front Microbiol. 2022 Jan 17;12:803307. doi: 10.3389/fmicb.2021.803307 (PMC8803141; doi:10.3389/fmicb.2021.803307)
Supplement: Supplementary file 2 [file Table_2.DOCX]

**Supplementary Table 2.** Genes whose deletion generated a significant loss or gain of fitness at only 30 minutes (important in early response to model honey) following model honey challenge.

| **Gene** | **Description** | **Operon structure** | **30’ (Significant)** | | **90’ (Not significant)** | |
| --- | --- | --- | --- | --- | --- | --- |
|  |  |  | **Log2 score** | **Adj. p value** | **Log2 score** | **Adj. p value** |
| ***Genes whose deletion caused loss of fitness on exposure to model honey*** | | | | | | |
| ***atpA*** | ATP synthase α subunit | *atpABCDEFGH* | -2.662 | 1.78×10^-12^ | -1.677 | 0.0434 |
| ***fabF*** | Fatty acid synthesis: 3-oxoacyl-ACP synthase 2 | *acpC-fabF* | -2.405 | 1.08×10^-16^ | -0.877 | 1 |
| ***moaC*** | Molybdopterin biosynthesis: Cyclic pyranopterin monophosphate synthase | *moaABCDE* | -2.254 | 1.41×10^-08^ | -0.854 | 1 |
| ***gntR*** | Transcription regulator | *gntR* | -2.156 | 3.37×10^-13^ | -1.079 | 0.2031 |
| ***moaE*** | Molybdopterin biosynthesis: Molybdopterin synthase catalytic subunit | *moaABCDE* | -2.116 | 1.26×10^-09^ | -1.325 | 0.1292 |
| ***modB*** | MoCo biosynthesis: Molybdenum transport system permease | *modABC* | -1.845 | 4.38×10^-06^ | -1.425 | 0.0340 |
| ***mogA*** | MoCo biosynthesis: Molybdenum cofactor biosynthesis protein | *mogA* | -1.778 | 2.40×10^-06^ | -0.837 | 1 |
| ***mrp*** | Iron-sulfur cluster carrier protein | *mrp* | -1.713 | 5.88×10^-09^ | -0.964 | 0.1264 |
| ***lon*** | Protease | *clpX-lon* | -1.562 | 0.0003 | -0.476 | 1 |
| ***ppk*** | Polyphosphate kinase | *ppk-ppx* | -1.363 | 3.71×10^-08^ | -0.950 | 0.2905 |
| ***glnA*** | Glutamine synthetase | *glnALG* | -1.225 | 0.0008 | -0.031 | 1 |
| ***gor*** | Glutathione reductase | *gor* | -1.007 | 0.0030 | -0.940 | 0.2791 |
| ***Genes whose deletion caused gain of fitness on exposure to model honey*** | | | | | | |
| ***phnl*** | Methylphosphonate degradation complex: carbon-phosphorus lyase core complex subunit | *phnCDEFGHIJKLMNOP* | 1.200 | 1.47×10^-05^ | -0.398 | 1 |
| ***phnF*** | Putative transcription regulator | *phnCDEFGHIJKLMNOP* | 1.245 | 5.67×10^-05^ | 0.136 | 1 |
| ***yagF*** | CP4-6 prophage; D-xylonate dehydratase | *yagEF* | 1.354 | 2.83×10^-11^ | -0.428 | 1 |
| ***ymjC*** | Uncharacterised protein | *ycjY-ymjDC-mpaA* | 2.722 | 0.0022 | N/A | N/A |

**Supplementary Table 3.** Genes whose deletion generated a significant loss or gain of fitness at only 90 minutes (important in adaptation to model honey) following model honey challenge.

| **Gene** | **Description** | **Operon structure** | **30’ (Not significant)** | | **90’ (Significant)** | |
| --- | --- | --- | --- | --- | --- | --- |
|  |  |  | **Log2 score** | **Adj. p value** | **Log2 score** | **Adj. p value** |
| ***Genes whose deletion caused loss of fitness on exposure to model honey*** | | | | | | |
| ***gnd*** | 6-phosphogluconase dehydrogenase | *gnd* | -0.538 | 1 | -1.172 | 0.0008 |
| ***yfjV*** | CP4-57 prophage; putative arsenite transporter | *yfjV* | -0.809 | 0.0642 | -1.173 | 0.0003 |
| ***yfjQ*** | CP4-57 prophage; DUF932 domain-containing protein | *yfjPQ* | -0.830 | 0.0671 | -1.254 | 0.0016 |
| ***yfeX*** | Porphyrinogen peroxidase | *yfeYX* | -0.920 | 1 | -1.396 | 0.0037 |
| ***Genes whose deletion caused gain of fitness on exposure to model honey*** | | | | | | |
| ***mgrR*** | Small regulatory RNA | *mgrR* | 0.166 | 1 | 2.187 | 0.0010 |
| ***yciF*** | DUF892 domain-containing protein | *yciGFE* | -0.900 | 0.1030 | 1.818 | 1.05×10^-05^ |
| ***yejG*** | Uncharacterised protein | *yejG* | 1.003 | 1 | 1.661 | 0.0037 |
| ***manA*** | Mannose-6-phosphate isomerase | *manA* | 0.523 | 1 | 1.429 | 0.0023 |
| ***ydcT*** | Putative ABC transporter ATP-binding protein | *ydcSTUV-patD* | 0.329 | 1 | 1.289 | 0.0026 |
| ***comR (ycfQ)*** | DNA-binding transcriptional repressor | *comR* | 0.422 | 1 | 1.263 | 0.0009 |
| ***plsX*** | Putative phosphate acyltransferase | *rpmF-plsX-fabHDG* | 0.520 | 1 | 1.209 | 2.73×10^-05^ |
| ***ydcC*** | H repeat-associated putative transposase | *ydcC* | 0.893 | 0.0242 | 1.165 | 3.08×10^-05^ |
| ***qseC*** | sensor histidine kinase | *qseBC* | 0.883 | 0.0103 | 1.112 | 0.0001 |
| ***ompC*** | Outer membrane porin | *ompC* | 0.624 | 0.5099 | 1.061 | 6.59×10^-07^ |
| ***mepS*** | Peptidoglycan DD-endopeptidase/peptidoglycan LD-carboxypeptidase | *mepS* | 0.619 | 1 | 1.013 | 0.0015 |

**Supplementary Table 4. List of primers**

| **Gene name** | **sequence** |
| --- | --- |
| *fdoH* | \| 5’-CCTTGTGAATGTGGAAAAGGTG-3’ \| \| --- \| \| 3’-ATGAAGCAGAAGGCGGTGAT-5’ \| |
| *fdhD* | \| 5’-CAGAATACCATAATGTTGGTGTGTG-3’ \| \| --- \| \| 3’-AGTCCTGCAATTTAATATATTTCGCAG-5’ \| |
| *moaA* | \| 5’-CCAGGGCGAAGGAAGAAATGAC-3’ \| \| --- \| \| 3’-GATAAATTCAGTGCTTACCTGACTCATCTG-5’ \| |
| *moeB* | \| 5’-GCGCTGGAAGATGGCAAATTACTG-3’ \| \| --- \| \| 3’-GACTTGAGCCATATAAGTTTGCAAG-5’ \| |
| *moaE* | \| 5’-GCGCTGGAAGATGGCAAATTACTG-3’ \| \| --- \| \| 3’-GACTTGAGCCATATAAGTTTGCAAG-5’ \| |
| *prc* | \| 5’-GATGCCACCGTATTAGAAATCACCAAAG-3’ \| \| --- \| \| 3’-GTAGCATCTGATTTACGGCATCTTGTCGCT-5’ \| |
| *gor* | \| 5’-GTAATCAACGATAAGGACACTTTG-3’ \| \| --- \| \| 3’-TTCTTATCTAACAATGAGACATGCG-5’ \| |
| o*mpR* | \| 5’-CTGGGCTACGTCTTTGTACC-3’ \| \| --- \| \| 3’-TATCGACGCCATCCTCAACG-5’ \| |
| *wecA* | \| 5’-GAATAAAGGTCTTCGTGGTTATACTTCTG-3’ \| \| --- \| \| 3’-CTCCACTCCTGACGAGCAAAAAACGTATA-5’ \| |
| *wecC* | \| 5’-ATAAGCAGCGAATTGTCGAGGAAGTGACG-3’ \| \| --- \| \| 3’-GATCGCAGATATCAACTTTCTCAAAGGC-5’ \| |
| *wecG* | \| 5’-GGCGCATGTCTGATGATCGCAAAACTGTTG-3’ \| \| --- \| \| 3’-GTGTTACCGGTTATCGTTATGGGTACATCG-5’ \| |
